# Deep learning-aided extraction of outer aortic surface from CT angiography scans of patients with Stanford type B aortic dissection

**DOI:** 10.1186/s41747-023-00342-z

**Published:** 2023-06-29

**Authors:** Risto Kesävuori, Tuomas Kaseva, Eero Salli, Peter Raivio, Sauli Savolainen, Marko Kangasniemi

**Affiliations:** 1 Department of Radiology, HUS Medical Imaging Center, Helsinki University Hospital and University of Helsinki, FI-00290 Helsinki, Finland; 2 Department of Cardiac Surgery, Heart and Lung Center, Helsinki University Hospital and University of Helsinki, Helsinki, Finland; 3 Department of Physics, University of Helsinki, Helsinki, Finland

**Keywords:** Computed tomography angiography, Convolutional neural network, Machine learning, Neural networks (computer), Aortic dissection

## Abstract

**Background:**

Guidelines recommend that aortic dimension measurements in aortic dissection should include the aortic wall. This study aimed to evaluate two-dimensional (2D)- and three-dimensional (3D)-based deep learning approaches for extraction of outer aortic surface in computed tomography angiography (CTA) scans of Stanford type B aortic dissection (TBAD) patients and assess the speed of different whole aorta (WA) segmentation approaches.

**Methods:**

A total of 240 patients diagnosed with TBAD between January 2007 and December 2019 were retrospectively reviewed for this study; 206 CTA scans from 206 patients with acute, subacute, or chronic TBAD acquired with various scanners in multiple different hospital units were included. Ground truth (GT) WAs for 80 scans were segmented by a radiologist using an open-source software. The remaining 126 GT WAs were generated via semi-automatic segmentation process in which an ensemble of 3D convolutional neural networks (CNNs) aided the radiologist. Using 136 scans for training, 30 for validation, and 40 for testing, 2D and 3D CNNs were trained to automatically segment WA. Main evaluation metrics for outer surface extraction and segmentation accuracy were normalized surface Dice (NSD) and Dice coefficient score (DCS), respectively.

**Results:**

2D CNN outperformed 3D CNN in NSD score (0.92 versus 0.90, *p* = 0.009), and both CNNs had equal DCS (0.96 versus 0.96, *p* = 0.110). Manual and semi-automatic segmentation times of one CTA scan were approximately 1 and 0.5 h, respectively.

**Conclusions:**

Both CNNs segmented WA with high DCS, but based on NSD, better accuracy may be required before clinical application. CNN-based semi-automatic segmentation methods can expedite the generation of GTs.

**Relevance statement:**

Deep learning can speeds up the creation of ground truth segmentations. CNNs can extract the outer aortic surface in patients with type B aortic dissection.

**Key points:**

• 2D and 3D convolutional neural networks (CNNs) can extract the outer aortic surface accurately.

• Equal Dice coefficient score (0.96) was reached with 2D and 3D CNNs.

• Deep learning can expedite the creation of ground truth segmentations.

**Graphical Abstract:**

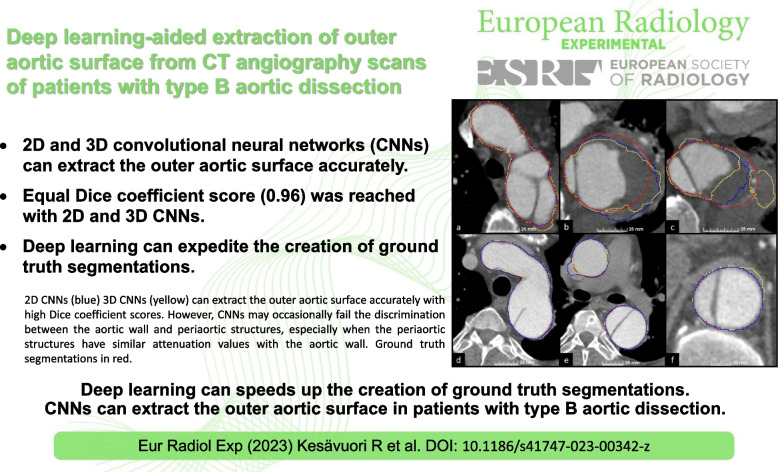

**Supplementary Information:**

The online version contains supplementary material available at 10.1186/s41747-023-00342-z.

## Background

Aortic dissections require prompt diagnosis and treatment to prevent aortic rupture and other major complications. In aortic dissection, blood enters the aortic wall through a tear in the inner layer of the aorta forming a false lumen inside the wall. Aortic dissections that do not involve the ascending aorta are classified as Stanford type B dissections (TBAD) [1, 2]. Follow-up imaging aims to find patients, who are at risk of developing complications. Reliable tools for aortic diameter measurements are required, because large diameter and fast growth rate of the aorta or false lumen are major risk factors for complications, and these factors guide surgical decision-making [1, 3]. Computed tomography angiography (CTA) is the primary imaging modality in aortic dissection, and aortic diameters should be measured perpendicular to its long axis using multiplanar reconstruction [1–5]. Manual aortic dimension measurements have suboptimal inter- and intra-rater reproducibility [3, 4, 6–13]. Therefore, observed aortic diameter changes of ≤ 3–5 mm during follow-up should be interpreted with caution [3].

**Fig. 1:**
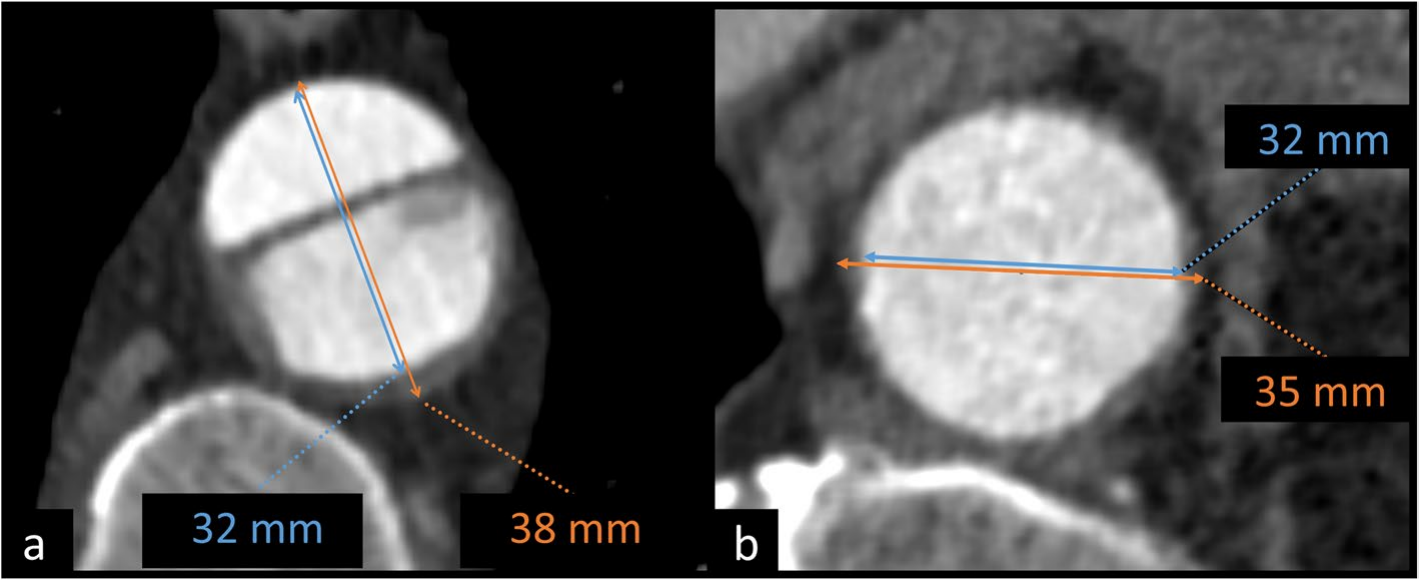
The differences between luminal (blue arrow) and outer-to-outer wall (orange arrow) dimension measurements. **a** TBAD patient with partly thrombosed false lumen. **b** Intact aorta of a TBAD patient with slight wall thickening at the level of diaphragm. *TBAD* Stanford type B aortic dissection

Aortic dimensions can be measured between the external (outer-to-outer wall) or internal (luminal) surfaces of the aortic wall, thus including or excluding the wall, respectively. On high-quality CTA scans, the aortic wall is often visible as a thin line encircling the contrast-enhanced lumen, as depicted in Fig. 1. The thickness of the aortic wall is typically from 1.5 to 2.5 mm, and it tends to thicken with aging [14, 15]. In aortic dissection, outer-to-outer wall measurements are preferred, because luminal measurements omit false lumen thrombosis and intramural hematoma (IMH) and may therefore underestimate the maximal diameter of the aorta, as depicted in Fig. 1 [2, 3, 5]. Recently published Society for Vascular Surgery and Society of Thoracic Surgeons reporting standards for type B aortic dissections and the 2010 American College of Cardiology Foundation/American Heart Association guidelines both recommend outer-to-outer wall dimension measurements in aortic dissection imaging [2, 5]. Similarly, a recent statement by American Heart Association recommends using the outer aortic contour as the landmark for aortic dimension measurements in patients with mural thrombus [3].

To enable automatic outer-to-outer wall aortic dimension measurements and volumetric analysis of the aorta, the whole aorta (WA) including the aortic wall needs to be segmented. Automatic segmentation of abdominal and thoracic aorta including the aortic wall has been studied previously [16–21]. While most of the works concentrating on the automatic segmentation of aortic dissection focus on the segmentation of true and false lumina [22–30], a few studies segmenting WA using convolutional neural networks (CNN) do exist [31–35]. However, the datasets in these studies are small, they comprise mainly of patients with other aortic diseases than aortic dissection, the imaging data is significantly preprocessed before CNN training, or the accuracy of outer aortic wall delineation in the manual segmentations is not described in detail [31–35].

In this study, we present a dataset consisting of 206 TBAD CTA scans with fully segmented ground truth (GT) WAs and high variation in TBAD imaging findings. The techniques and challenges faced in the manual and semi-automatic segmentation of WAs are discussed. Two-dimensional (2D) and three-dimensional (3D) CNNs are trained to segment the WAs automatically and evaluated both on the tasks of segmentation of WA and extraction of the outer aortic surface.

## Methods

The Helsinki University Hospital’s ethical committee approved this retrospective study, and patients’ informed consent was waived.

### Dataset and segmentation process

A total of 240 consecutive patients diagnosed with TBAD at our institution from January 2007 to December 2019 were retrospectively reviewed for this study. Patients, who did not have any aortic CTA scans or who had only > 3-mm axial reformats available in our institution’s picture archiving and communication systems, were excluded from this study. From each patient, one CTA scan with TBAD imaging findings was included in the dataset. In an aim to include more complex imaging findings of TBAD to the dataset, the latest available CTA scan was often selected, because false lumen thrombosis and aneurysmal degeneration usually develop over time [3]. This resulted in a dataset of 206 aortic CTA scans with diverse TBAD imaging findings acquired between 2007 and 2020.

**Fig. 2:**
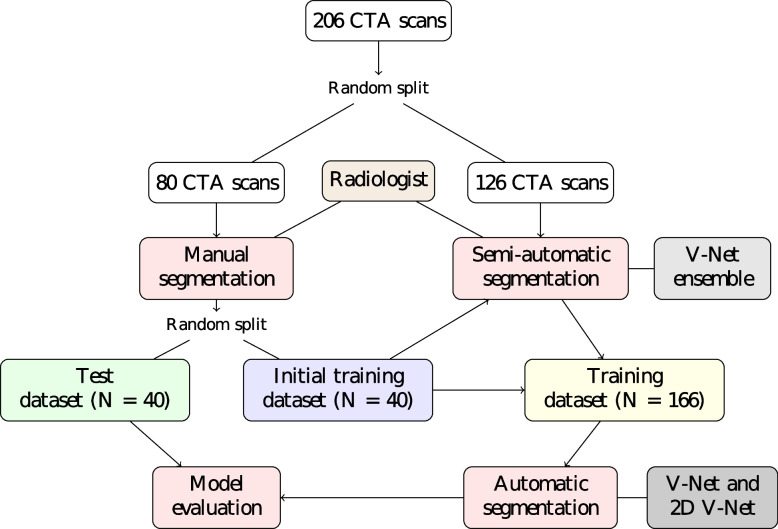
Diagram of the study: 206 CTA scans from 206 patients with TBAD were split randomly to subsets of 80 and 126 scans. Ground truth WAs from the first subset were manually segmented by a radiologist using 3D Slicer. GT WAs of the second subset were generated via a semi-automatic segmentation process. In this process, initial training set, consisting of randomly chosen 40 scans from the first subset with manually segmented WAs, was utilized to train V-Net ensemble. The ensemble produced initial segmentations for the second subset. These segmentations were corrected by the radiologist and combined with the initial training set to form a training set of 166 studies. V-Net and 2D V-Net were trained using the training set and evaluated over the test set of 40 studies on tasks of automatic segmentation of WAs and extraction of the outer walls. *CTA* Computed tomography angiography, *TBAD* Stanford type B aortic dissection, *WA* Whole aorta

In order to expedite the segmentation process, GT WAs for the CTA scans were generated in two phases as described in Fig. 2:

- (1) Manual segmentation: A radiologist manually segmented 80 scans chosen randomly from the 206 scans; the segmented scans were randomly divided to form a test set and an initial training set with 40 scans in each set.
- (2) Semi-automatic segmentation: The initial training set was used to train a 3D CNN ensemble, which automatically produced preliminary segmentations for the remaining 126 scans; these segmentations were subsequently corrected by the radiologist. The final dataset was then formed including 40 scans for testing and 166 scans for training.

### Manual segmentation

Manual WA GT segmentations were created for 40 scans in the test set and 40 scans in the training set. The external aortic wall surface contours from the aortic valve to the common iliac arteries were annotated in every axial slice of each image volume by a radiologist with over 5 years of experience in aortic imaging to create the WA segmentations. The annotations were performed using 3D Slicer’s Segment Editor module [36]. The outer aortic wall surface contours were drawn using a commercially available pen display (Wacom Cintiq Pro 16, Wacom Co. Ltd., Kazo, Saitama, Japan, or Microsoft Surface Pro 8, Microsoft Corporation, Redmond, WA, USA) to produce robust WA segmentations. When the aorta was surrounded by fat tissue, intensity-based drawing methods were used when appropriate to include the outermost aortic wall voxels (higher density than fat) to the WA segmentations. Coronal and sagittal reformats or multiplanar reconstruction was used to confirm precise annotation of the external aortic wall surface when necessary. Special attention was paid to drawing of aortic segments that run along with denser structures (*e.g.*, diaphragm, pleura, or inferior vena cava) to reliably exclude periaortic structures from the segmentations. Coronary, supraaortic, or visceral arteries were not included in the segmentations. Supraaortic arteries were segmented to the level of branching from the aorta in the axial plane. At the coronary and visceral artery ostia, the WA segmentations were interpolated as a continuation of the outer aortic surface. Aortic wall calcifications were included in the WA segmentations. After completing the manual segmentation process, the 40 test set scans were re-examined, and their GTs were corrected if necessary. The average duration of manual and semi-automatic segmentations was estimated based on time used to complete 10 consecutive segmentations including file saving and opening of a new CTA scan for segmentation.

**Fig. 3:**
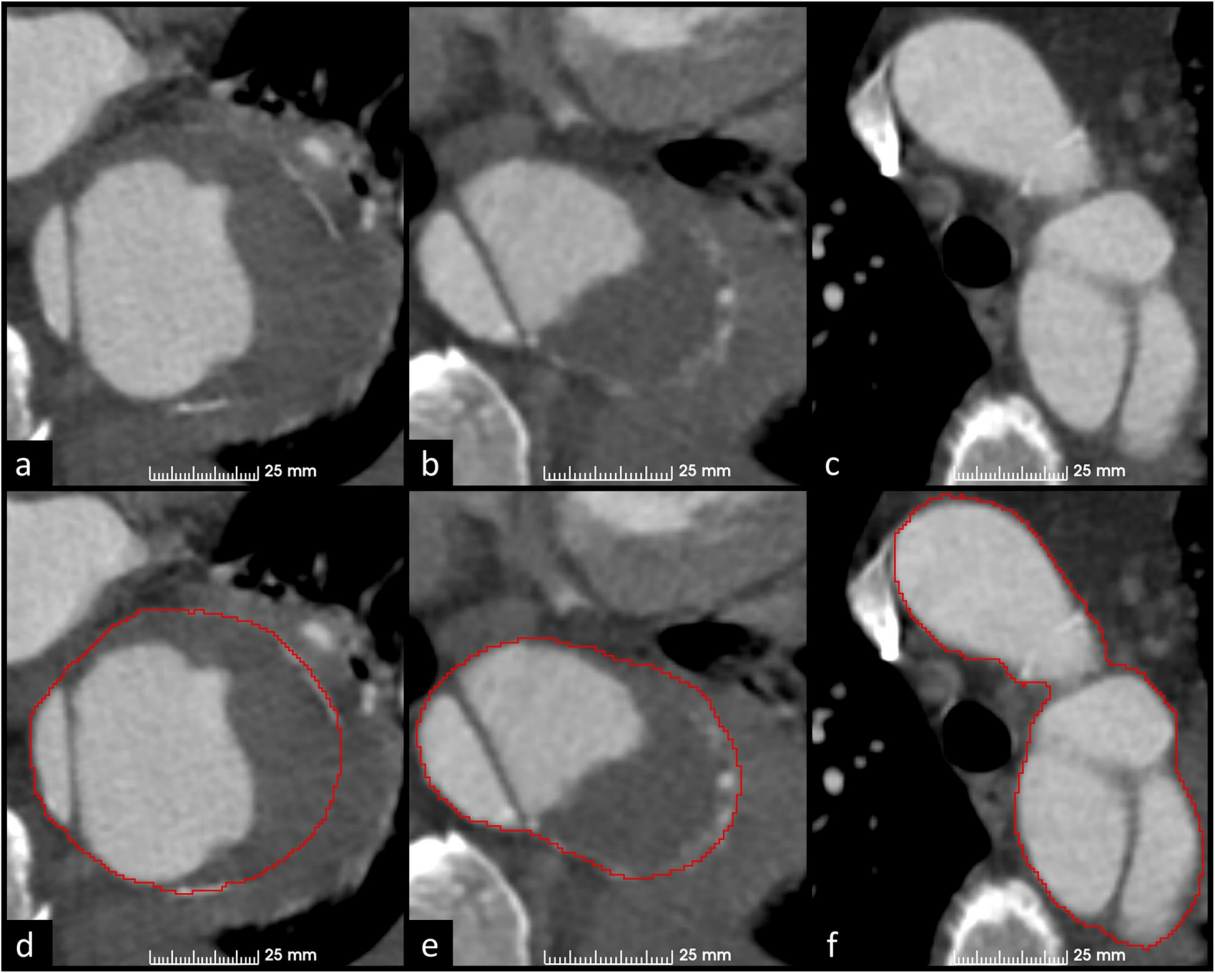
Manual WA segmentations of a patient with pleural fluid and false lumen thrombosis at the level of descending aorta (**a**, **b**, **d**, and **e**) and aortic arch (**c**, **f**). Delineation of the outer aortic surface is obscured due to similar intensities of pleural fluid and false lumen thrombosis. Multiplanar reconstruction was used to precisely annotate the outer aortic surface margin. *WA* Whole aorta

**Fig. 4:**
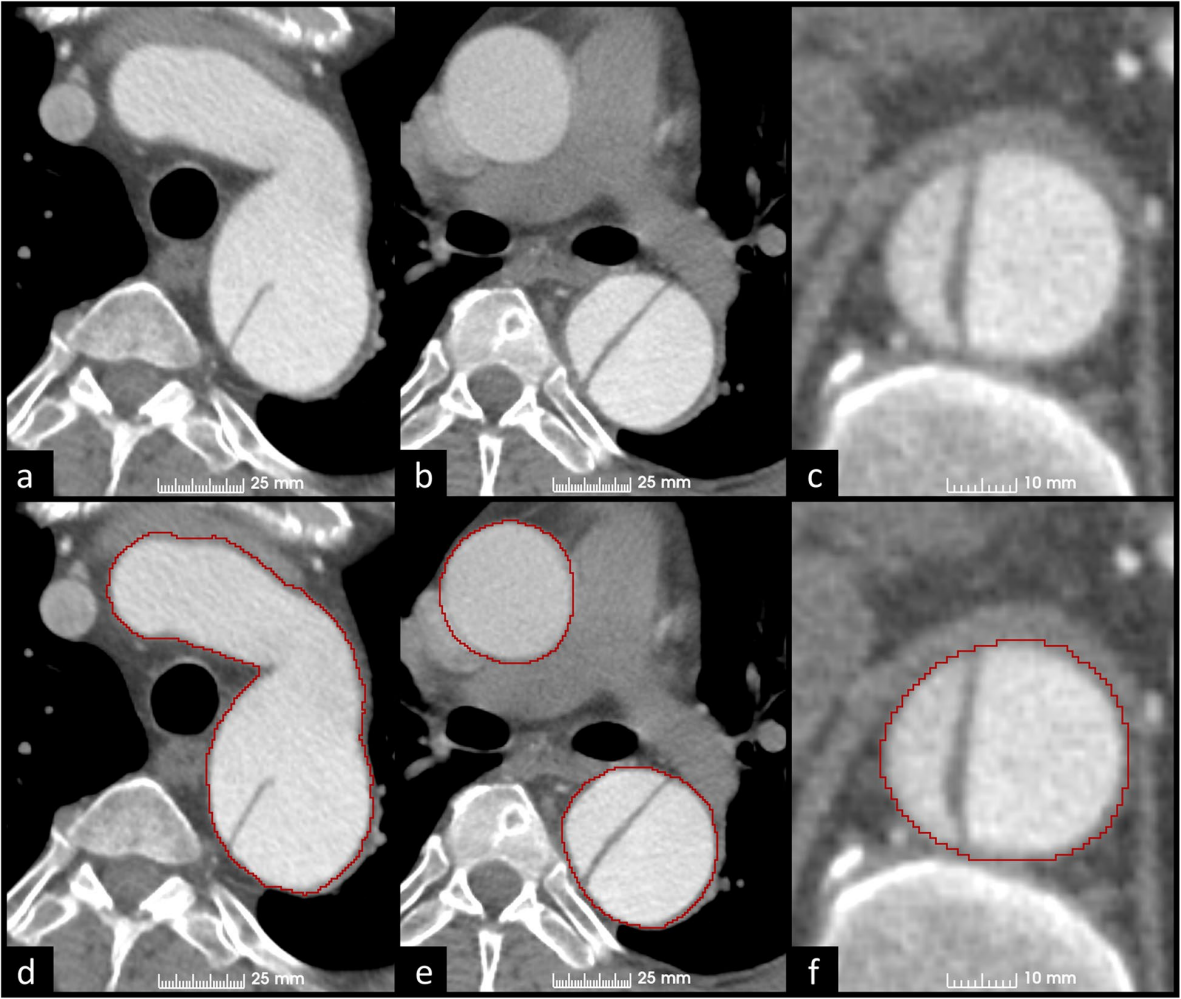
Manual WA segmentation of a patient with TBAD at the level of aortic arch (**a**, **d**), ascending aorta (**b**, **e**), and diaphragm (**c**, **f**). Notice the motion artifact in the ascending aorta due to non-ECG-gated imaging obscuring the aortic surface margins and leading to imperfect outer surface annotation. *ECG* Electrocardiogram, *TBAD* Stanford type B aortic dissection, *WA* Whole aorta

Figures 3 and 4 present examples of manual GT segmentations of two patients with different false lumen morphologies. In Fig. 3, false lumen thrombosis and pleural fluid have nearly similar intensity values, which complicates the extraction of the outer aortic surface (Fig. 3a and d). Coronal and sagittal reformats were used to confirm correct outer surface annotation. Figure 4 describes more simple segmentation task in a TBAD patient with open false lumen, but motion artifact in ascending aorta obscures the aortic surface margins and leads to imperfect outer surface annotation (Fig. 4b and e).

### Semi-automatic segmentation

Forty manually created WA GT segmentations were used to form the initial training set. Then, five different V-Net models were trained by dividing the initial training set to five unique folds with each fold having 32 training and eight validation scans. Training was performed using MONAI, an open-source framework based on PyTorch [37, 38]. The architecture of each V-Net was the same and followed the default V-Net configuration given in the framework. This architecture was based on the one introduced by Milletari et al. [39] but included batch normalization before each parametric rectified linear unit activation function, dropout before each encoder and decoder block, and another dropout applied on the forwarded fine-grained features. The dropout probability of both dropouts was 0.5.

The patch size was 192 × 192 × 48 voxels. Prior to the extraction of the patches, the intensities of the volumes were scaled from [-50, 600] to [0, 1] with clipping the values outside the initial range to the output range. Spacings were normalized to 1.0 × 1.0 × 3.0 mm. Batch size was 8 with 4 patches collected from two different scans. In each batch, the patches were randomly chosen, but so that half of the patches had centers as foreground voxels and the other half centers as background voxels. The optimizer was Adam and the learning rate 0.0001. The loss function of each model consisted of summation of Dice coefficient score (DCS) and binary cross entropy. The total number of iterations was 16,000. Validation was performed every 128 iterations, and the validation metric was average DCS over all image volumes in the validation set. The model configuration with the highest validation metric was chosen as the final model.

The models performed inference on the 126 scans which were left out from the manual segmentation process. For each scan, five different predictions from each model were generated with sliding window inference technique using the same patch size as in training and 25% overlap. The predictions were binarized by applying the argmax operation on the output of the model. After the binarization, value 1 defined aortic voxels and value 0 other voxels.

Five different ensemble segmentations were formed using these binarizations: In the ensemble N, a voxel was marked as aorta voxel if it belonged to aorta at least in N of the binarized predictions. Thus, in the ensemble 1, the aorta was the combination (union) of all five predictions, ensemble 3 was the majority voting ensemble, and in the ensemble 5, only the aorta voxels that overlapped in all five predictions were marked to aorta. One ensemble segmentation of each scan was visually chosen to be manually corrected. In most cases, majority of voting ensemble was chosen, but occasionally, another ensemble with more suitable predictions was selected. The segmentations were preliminary corrected by a researcher with 1 year of experience on the manual aortic segmentation process and subsequently corrected by the radiologist with experience of aortic imaging using 3D Slicer’s Segment Editor module. The corrections were performed with similar methods and robustness as manual segmentations.

### Automatic segmentation

Two neural network models, 2D V-Net and V-Net, were trained for automatic segmentation of the WA. The input of the first consisted of 320 × 320 sized axial slices, whereas the input of the second is of 224 × 224 × 64 sized patches. V-Net had the same architecture as discussed in semi-automatic segmentation, but the number of filters of V-Net started from 12 instead of 16 proposed in Milletari et al. [39]. 2D V-Net was simply a 2D version of the V-Net model used in the semi-automatic segmentation. The dropout probabilities for both models were set as 0.4. Both models had the same 136 scans used for training and 30 scans used for validation. The validation set was chosen randomly.

Before extracting the inputs, the spacings of image volumes were normalized similarly as in semi-automatic segmentation, but the scaling was performed from [− 500, 1,500] to [0, 1]. The batch size was 32 for 2D V-Net with 16 slices extracted from two scans. The size was 4 for V-Net with 2 patches extracted from two scans. In each batch, the choice of samples was performed similarly as in the semi-automatic segmentation. The optimizer and the loss function were also the same as used in the semi-automatic segmentation. The learning rate was 0.001 and halved after 81,600 iterations. The total number of iterations was 136,000 for 2D V-Net and 125,800 for V-Net. During training, axial rotations up to 45° were performed to every second sample of the batch randomly as augmentation. The validation process was the same as explained in the semi-automatic segmentation but performed after every 3,400 iterations for both 2D V-Net and V-Net.

### Model evaluation

Both 2D V-Net and V-Net performed inference on the scans in the test set using the same sliding window technique utilized in semi-automatic segmentation. The patch size of V-Net was 416 × 416 × 64 and the dimensions of the axial slices of 2D V-Net 416 × 416. The evaluation metrics used were DCS, Hausdorff distance (HD, mm), normalized surface Dice (NSD), and mean surface distance (MSD, mm) [40]. The HD was calculated using 95th percentile of the distances. DCS is a measure of the overlap between the prediction and the GT segmentations, HD measures the maximum distance between the surfaces of segmentations, and MSD measures the average distance. NSD measures overlap between the surfaces of the prediction and GT. The aorta voxels which had at least one non-aorta voxel in the first-order 3D neighborhood were defined as surface voxels. The tolerance parameter of the NSD specifies the maximum difference in the boundary that is tolerated without penalty in the metric. For NSD, 1-mm tolerance level was selected. Results are reported as median values and interquartile ranges calculated over the studies. Wilcoxon signed-rank test for the zero median for the difference of paired samples of V-Net and 2D V-Net was conducted, and corresponding *p*-values were calculated.

## Results

The datasets and demographic characteristics are presented in Table 1, and the performance scores of the models are presented in Table 2, which includes the medians and interquartile ranges of the evaluation metrics computed over the test set with V-Net and 2D V-Net; *p*-values of each metric between the models are also illustrated. The median NSD scores of V-Net and 2D V-Net were 0.90 and 0.92, median HD scores 2.22 mm and 2.36 mm, median DCS 0.96 and 0.96, and median MSD scores 0.43 mm and 0.44 mm, respectively. Only the difference between NSD scores of the models was statistically significant. All metrics evaluating surface delineation, *i.e.*, NSD, HD, and MSD, have relatively high dispersion in values.

**Table 1:** Description of the datasets

	Training dataset (*n* = 166)	Test dataset (*n* = 40)	*p*-value*
Age, years	71 (59–79)	66 (53–74)	0.049
Male, *n* (%)	49 (30)	9 (23)	0.438
Hyperacute dissection (initial CT), *n* (%)	53 (32)	12 (30)	0.852
Acute or subacute dissection (1–90 days), *n* (%)	47 (28)	13 (33)	0.698
Chronic dissection (> 90 days), *n* (%)	66 (40)	15 (38)	0.858
Intraluminal or intramural thrombosis in at least two aortic zones**, *n* (%)	134 (81)	31 (78)	0.662
Classic dissection, *n* (%)	26 (62)	27 (68)	0.716
IMH, *n* (%)	60 (36)	13 (33)	0.716
CT-scanner vendor, *n* (%)			
Siemens	134 (81)	31 (78)	0.662
GE Medical Systems	26 (16)	7 (18)	0.812
Toshiba	6 (4)	2 (5)	0.654

Data is presented as counts (percentages) or median (interquartile range). *Fisher’s exact test and Mann–Whitney *U*-test for categorical and continuous variables, respectively. **Lombardi et al. (J Vasc Surg 2020, reference [5]). *CT* Computed tomography, *IMH* Intramural hematoma

**Table 2:** Evaluation scores of V-Net and 2D V-Net over the test set

Model	NSD	HD (mm)	DCS	MSD (mm)
V-Net	0.90 (0.84–0.93)	**2.22** (1.68–3.56)	0.96 (0.95–0.97)	**0.43** (0.31–0.61)
2D V-Net	**0.92** (0.85–0.94)	2.36 (1.49–4.12)	0.96 (0.95–0.97)	0.44 (0.29–0.72)
*p*-value*	0.009	0.614	0.110	0.125

Each score is presented as median (interquartile range). With NSD and DCS, higher is better, while with MSD and HD, lower is better. The best scores are bolded. *Wilcoxon signed-rank test for the zero median for the difference of paired samples of V-Net and 2D V-Net. *DCS* Dice coefficient score, *HD* 95th percentile Hausdorff distance, *MSD* Mean surface distance, *NSD* Normalized surface dice

**Fig. 5:**
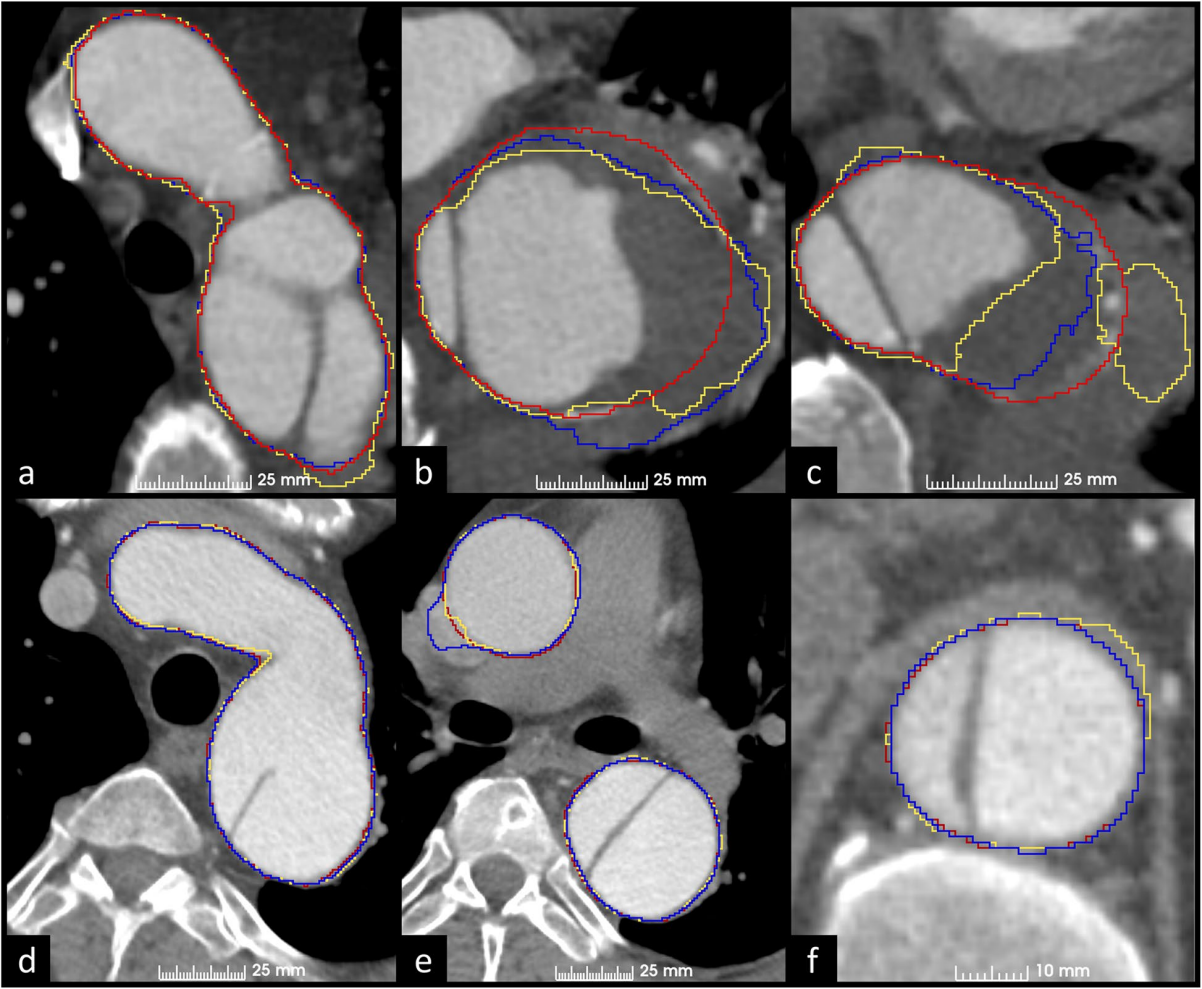
Examples of automatic segmentations of 2D V-Net (blue) and V-Net (yellow) and the corresponding manual ground truth segmentations (red) on the same axial slices than in Figs. 3 and 4. Both 2D V-Net and V-Net falsely label pleural fluid as WA and omit part of false lumen thrombosis from WA segmentations (**a**, **b**, **c**). Both 2D V-Net and V-Net correctly omit atelectatic lung from WA segmentations (**d**). Motion artifact in the ascending aorta complicates automatic segmentation (**e**); note the correct automatic segmentation of the descending aorta at this level. V-Net labels part of the diaphragm as WA, while 2D V-Net correctly labels the aorta (**f**). *WA* Whole aorta

**Fig. 6:**
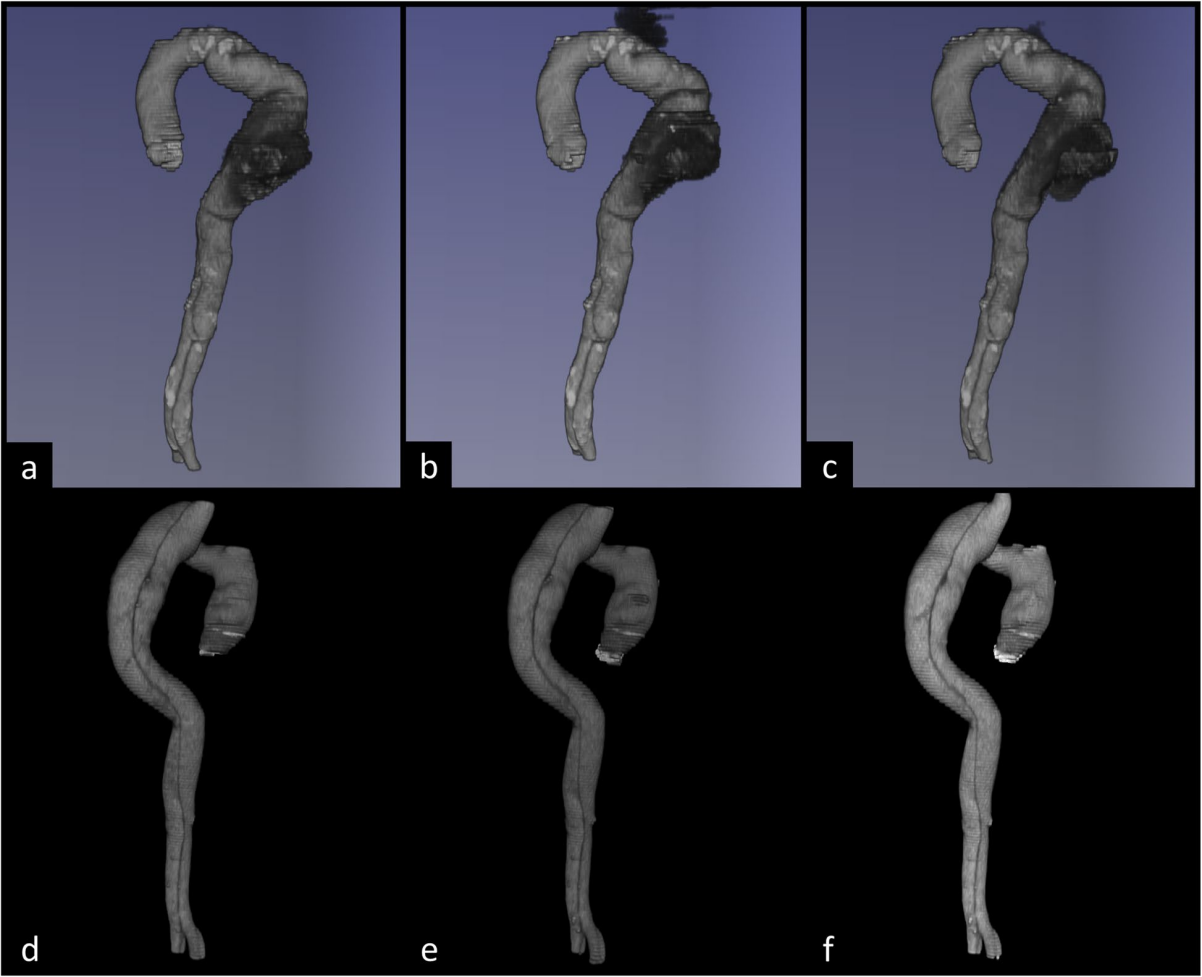
Three-dimensional representations of ground truth (**a**, **d**), 2D V-Net (**b**, **e**), and V-Net (**c**, **f**) segmentations of two different patients (**a**–**c**, patient from Fig. 3; **d**–**f**, patient from Fig. 4). CNNs variably detect the border between thrombosed false lumen and pleural fluid (**b**, **c**). High agreement between ground truth and CNN segmentations (**d**–**f**). *CNN* Convolutional neural network

In Fig. 5, we present the same axial slices as in Figs. 3 and 4 with GT, V-Net, and 2D V-Net segmentations. Both 2D V-Net and V-Net fail the discrimination between thrombosed false lumen and pleural fluid (Fig. 5b and c). Both CNNs have difficulties delineating ascending aorta with motion artifact (Fig. 5e), whereas V-Net mistakenly adds part of the diaphragm as WA (Fig. 5f). NSD scores over the scan of the first row (Fig. 5a, b, c) were 0.79 with V-Net and 0.73 with 2D V-Net. NSD score over the scan presented in the second row (Fig. 5d, e, f) was 0.96 with both V-Net and 2D V-Net. In Fig. 6, we present the GT, 2D V-Net, and V-Net segmentations in 3D.

On average, the duration of manual segmentation of one scan ranged from 45 to 75 min, and correction of a semi-automatic segmentation lasted between 20 and 40 min. The segmentation times included loading of the scan and saving the segmentation using 3D Slicer. On occasional poor automatic segmentations, the duration of semi-automatic segmentation was comparable to manual segmentation times. The training time of V-Net and 2D V-Net was 160 h and 16 h, respectively, using Tesla V100 GPU. The inference times of the models were on average less than 1 min.

## Discussion

This study presented a dataset of 206 CTA scans from 206 patients with fully segmented GT WAs of TBAD including the aortic wall. The study proposed deep learning-based semi-automatic segmentation process to expedite the segmentation process and evaluated the use of 2D- and 3D-based CNNs for segmentation of WA and the extraction of the outer aortic wall surface. Semi-automatic segmentation process was approximately twice as fast as manual segmentation process. 2D and 3D CNNs reached 0.92 and 0.90 median NSD scores over the test set of 40 scans, respectively. The results obtained do not justify automation of WA segmentation but suggest that CNNs can be helpful in semi-automatic segmentation of WA and outer aortic wall extraction.

In this study, we introduce a large and robustly segmented WA dataset with diverse TBAD imaging findings. Previously, Cao et al. [31] presented a dataset of 276 CTA scans with WA, true lumen, and false lumen GT labels. Their dataset comprised of TBAD patients undergoing TEVAR (thoracic endovascular aortic repair), and the segmentations were produced to meet the requirements for its planning and standard TBAD measurements. The authors segmented WA GTs in intervals and used fill-between-slices tool to fill the gaps between slices. We approached the segmentation process from a different perspective. Our aim was to produce accurate annotations of the outer surface of the aorta in every axial slice in a patient population with diverse set of aortic dissection imaging findings to enable robust dimension measurements of a dissected aorta in the future. The WA dataset of Bratt et al. [32] consisted of over 5,000 scans with various aortic pathologies, including aortic dissections, but the authors explicitly mentioned that the GTs were not originally generated for dimension measurements. Sieren et al. [35] studied automatic segmentation of WAs of healthy and diseased aortas but did not describe how accurately they aimed to delineate the aorta’s outer surface. Krissian et al. [33] aimed to carefully segment WAs in their work but segmented only five scans. Wobben et al. [34] reported high DCSs of TL, FL, FL thrombosis, and aorta segmentations using deep learning using preprocessed imaging data for CNN training. The data consisted of 80 × 80 mm cropped multiplanar reconstructions of the aorta centered along and perpendicular to the aortic centerline. Yu et al. [22] and Yao et al. [23] discussed results on WA segmentation, but in these works, the wall of the aorta was not included in the WA segmentations.

In previous studies by Sieren et al. [35] and Bratt et al. [32], WAs were manually segmented in 30 min on average. In our material, manual segmentation time of one scan was on average 1 h despite using 3-mm axial slices in contrast to Sieren et al. [35] who used 1-mm axial slices in the segmentation process. Sieren et al. [35] and Bratt et al. [32] used semi-automatic tools in their segmentation processes, which may explain the difference in the segmentation times.

We opted not to use semi-automatic tools in the manual segmentation process, since this could have resulted in segmentation imprecisions. Instead, we opted to segment manually 80 scans of our dataset and use deep learning-based semi-automatic segmentation process in the creation of GTs for the remaining 126 scans. The aim of this process was to reduce the manual annotation time used in the creation of GTs. In the process, initial segmentations were first generated via an ensemble of V-Nets and then corrected manually. The use of deep learning as an aiding tool in the creation of GTs has been studied previously [41]. We chose to use the ensemble to effectively utilize the 40 manually segmented GTs of the initial training set. Via ensembling, all 40 studies of the set could be utilized for both training and validation. The ensemble achieved an average of 0.94 DCS over the ensemble validation sets. In large parts of the aorta, the initial segmentations followed the outer aortic margin correctly, and manual corrections were performed only in the areas with imprecise initial segmentations. The process decreased the segmentation time approximately by half but maintained the quality of the annotations. The test set was segmented manually without using semi-automatic process to ensure that the test set was independent of the training set.

Previous studies have evaluated the performance of automatic WA segmentation methods primarily with DCS metrics, with reported mean DCSs of 0.91 to 0.96 [31–33, 35]. The datasets in these studies have varied in terms of aortic pathology, WA segmentation accuracy, and area of the aorta used for DCS calculation. In only one study, by Cao et al. [31], the dataset has comprised solely of patients with aortic dissection where 0.93 DCS was reached. In our study, 2D V-Net and V-Net achieved 0.96 median DCSs, which is a good agreement considering the variability of TBAD imaging findings in our dataset and high occurrence of motion artifacts at the level of the aortic root and ascending aorta. The performance of the models on the task of extraction of outer aortic surface was quantified using NSD, HD, and MSD scores. With NSD, we selected a tolerance threshold of 1 mm, because contemporaneous 1-mm segmentation errors in opposite sides of the aorta would lead to a 2-mm error in dimension measurements. Such error was considered unacceptable for clinical use.

Median NSD scores of 2D V-Net and V-Net were 0.92 and 0.90. The difference between the scores was deemed statistically significant, while no significant difference was observed between the other scores. Consequently, V-Net was unable to outperform 2D V-Net in this study, although the CTA scans used were inherently 3D. In addition to our study, at least Bratt et al. [32] have reported the applicability of 2D CNNs on the task of segmenting 3D WAs. The usage of 2D CNNs is intriguing since they are significantly faster to train than the 3D CNNs. Through the example shown in Fig. 5, we showed that segmentations with NSD scores as high as 0.96 could include unacceptable mistakes. Thus, the median NSD scores of models are yet too low to justify automation of the outer aortic wall extraction. Most major segmentation mistakes by both CNNs were related to unsuccessful delineation between false lumen thrombosis or IMH and periaortic structures with near similar intensity values, such as pleural fluid, diaphragm, or large hiatal hernias. On the other hand, the models can be very useful in semi-automatic setting. Sieren et al. [35] reported a 0.99-mm median MSD score on the WA border extraction over a subgroup of scans with patients suffering from aortic dissection. The same score over our test set was 0.44 mm with 2D V-Net and 0.43 mm with V-Net. While the datasets are different, this result comparison highlights the suitability of V-Net type CNN architectures for outer aortic surface extraction.

Evaluation of aortic growth during follow-up and imaging-based risk assessment for aortic rupture after aortic dissection is based on dimension measurements in several areas of the aorta and lumina [1, 3]. The aim of this work was to produce a robust automatic CNN-based method for WA segmentation and to evaluate the segmentation task in detail. The CTA scans and the WA segmentations included variable presentations of aortic, true lumen, and false lumen morphologies, intraluminal thrombosis, and IMH. In the future, we aim to combine our WA segmentation approach with lumen segmentations and proceed to compute the measurements.

This is a retrospective study with limitations. Although the GT segmentations in this study were performed by a radiologist experienced in aortic imaging, the outer wall surface annotations in the datasets may occasionally be imperfect. Additionally, we did not evaluate GT segmentation quality or agreement between radiologists. Albeit we included a large diversity of aortic dissection CTA scans with varying morphologic findings, imaging parameters, and image quality, a larger training set would have produced a more thorough set of aortic dissection imaging findings. Therefore, rare findings in the test dataset not presented at the training set were not adequately labeled by the final neural network.

In conclusion, we introduced a novel, diverse dataset of WA segmentations and evaluated deep learning-based approaches on the automatic generation of the segmentations. We adduced the difficulty of manual WA segmentation process and illustrated that the use of deep learning as a part of a semi-automatic segmentation pipeline can speed up the process without compromising segmentation quality. We reached promising results on the automatic segmentation task using both 3D- and 2D-based CNN models.

## Supplementary Information

Additional file 1.

## Data Availability

The datasets used and analyzed during the current study are available from the corresponding author on reasonable request.

## Abbreviations

2D: Two dimensional
3D: Three dimensional
CNN: Convolutional neural network
CT: Computed tomography
CTA: Computed tomography angiography
DCS: Dice coefficient score
GT: Ground truth
HD: Hausdorff distance
IMH: Intramural hematoma
MSD: Mean surface distance
NSD: Normalized surface dice
TBAD: Stanford type B aortic dissection
WA: Whole aorta
