# Supplementary material for: Deep learning-aided extraction of outer aortic surface from CT angiography scans of patients with Stanford type B aortic dissection
Source: Eur Radiol Exp. 2023 Jun 29;7:35. doi: 10.1186/s41747-023-00342-z (PMC10307760; doi:10.1186/s41747-023-00342-z)
Supplement: Supplementary file 1 — Additional file 1. [file 41747_2023_342_MOESM1_ESM.pdf]

ELECTRONIC SUPPLEMENTARY MATERIAL

Deep learning-aided extraction of outer aortic surface from CT angiography scans of patients with Stanford type B aortic dissection

Supplementary Table 1. CT-scanner manufacturers, models, and imaging parameters of the 206 CT-studies included in this study. CT-scans were performed with different CT-scanners and varying imaging parameters in several different hospital units of our hospital district between the years 2007 and 2020.

Manufacturer	Model	Count (n)	Pixel spacing (mm)	Kernel (n)	kVp (kV)	Imaging delay after contrast injection (s)	Contrast volume (ml)	Contrast flow rate (ml/s)	Contrast flow duration (s)
SIEMENS	SOMATOM Definition AS+	72	0.8086 (0.5703-0.9766)	I26f (49)<br>B30f (16)<br>I30f (6)<br>I31f (1)<br>T20f (1)	100 (80-140)	67.2<br>(24.5-108.4)	60.0 (59.6-120.7)	4.9 (3.8-5.0)	12.3 (12.0-15.4)
	SOMATOM Definition Flash	59	0.7344 (0.6484-0.9766)	I26f (59)	100 (80-140)	68.1 (18.2-90.6)	70.0 (34.3-110.3)	4.6 (2.4-5.0)	15.0 (8.0-22.5)
	SOMATOM Definition Edge	31	0.8281 (0.5801-0.9766)	I26f (25)<br>B26f (6)	120 (100-140)	75.1 (19.5-94.6)	80.1 (39.8-110.7)	4.9 (3.2-5.0)	18.1 (12.0-25.1)
	SOMATOM Sensation 4	2	0.6387 (0.5352-0.7422)	B30f (2)	120 (120-120)				
	SOMATOM go.Top	1	0.6103 (0.6103-0.6103)	Bv36f (1)	110 (110-110)				
	SOMATOM Emotion 6	1	0.6172 (0.6172-0.6172)	B31s (1)	110 (110-110)				
GE MEDICAL SYSTEMS	LightSpeed VCT	13	0.8145 (0.5938-0.9766)	SOFT (13)	100 (80-120)				
	Revolution EVO	6	0.6963 (0.6426-0.7637)	SOFT (6)	100 (80-100)				
	Discovery CT750 HD	5	0.7500 (0.5625-0.9102)	STANDARD (5)	100 (80-100)				
	LightSpeed Ultra	5	0.7031 (0.6836-0.8066)	STANDARD (5)	120 (120-120)				
	HiSpeed QX/i	1	0.7012 (0.7012-0.7012)	STANDARD (1)	120 (120-120)				
	LightSpeed Pro 32	1	0.7988 (0.7988-0.7988)	STANDARD (1)	100 (100-100)				
	Revolution CT	1	0.9766 (0.9766-0.9766)	STANDARD (1)	100 (100-100)				
	Revolution HD	1	0.9023 (0.9023-0.9023)	SOFT (1)	100 (100-100)				
TOSHIBA	Aquilion PRIME	3	0.8160 (0.8160-0.8160)	FC08 (2)<br>FC18 (1)	100 (100-100)	26.0 (24.4-64.7)			
	Aquilion	3		FC03 (3)	120 (120-120)	30.2 (24.9-35.5)			
	Aquilion ONE	1		FC08	100 (100-100)	69.4 (69.4-69.4)			

Numerical data are presented as median (range). kVp = Kilovoltage peak.
